# Efficient Synthesis of a Maghemite/Gold Hybrid Nanoparticle System as a Magnetic Carrier for the Transport of Platinum-Based Metallotherapeutics

**DOI:** 10.3390/ijms16012034

**Published:** 2015-01-16

**Authors:** Pavel Štarha, David Smola, Jiří Tuček, Zdeněk Trávníček

**Affiliations:** 1 Regional Centre of Advanced Technologies and Materials, Department of Inorganic Chemistry, Faculty of Science, Palacký University, 17. listopadu 12, Olomouc CZ-77146, Czech Republic; E-Mails: pavel.starha@upol.cz (P.S.); david.smola01@upol.cz (D.S.); 2 Regional Centre of Advanced Technologies and Materials, Department of Experimental Physics, Faculty of Science, Palacký University, 17. listopadu 12, Olomouc CZ-77146, Czech Republic; E-Mail: jiri.tucek@upol.cz

**Keywords:** nanoparticles, maghemite, magnetic, cisplatin, drug delivery

## Abstract

The preparation and thorough characterization of a hybrid magnetic carrier system for the possible transport of activated platinum-based anticancer drugs, as demonstrated for cisplatin (*cis*-[Pt(NH_3_)_2_Cl_2_], CDDP), are described. The final functionalized mag/Au–LA–CDDP* system consists of maghemite/gold nanoparticles (mag/Au) coated by lipoic acid (HLA; LA stands for deprotonated form of lipoic acid) and functionalized by activated cisplatin in the form of *cis*-[Pt(NH_3_)_2_(H_2_O)_2_]^2+^ (CDDP*). The relevant techniques (XPS, EDS, ICP-MS) proved the incorporation of the platinum-containing species on the surface of the studied hybrid system. HRTEM, TEM and SEM images showed the nanoparticles as spherical with an average size of 12 nm, while their superparamagnetic feature was proven by ^57^Fe Mössbauer spectroscopy. In the case of mag/Au, mag/Au–HLA and mag/Au–LA–CDDP*, weaker magnetic interactions among the Fe^3+^ centers of maghemite, as compared to maghemite nanoparticles (mag), were detected, which can be associated with the non-covalent coating of the maghemite surface by gold. The pH and time-dependent stability of the mag/Au–LA–CDDP* system in different media, represented by acetate (pH 5.0), phosphate (pH 7.0) and carbonate (pH 9.0) buffers and connected with the release of the platinum-containing species, showed the ability of CDDP* to be released from the functionalized nanosystem.

## 1. Introduction

Targeted drug delivery involving nanoparticles (e.g., iron oxides or gold) is currently generally accepted as a suitable and prospective alternative pathway in conventional anticancer chemotherapy using platinum-based metallotherapeutics [1,2,3]. The basic advantage is that the targeted delivery could reduce the amount of drug administrated to the body, and as a consequence of this, the negative side effects (e.g., nephrotoxicity, neurotoxicity or myelosuppression) can be partially eliminated [4]. Additionally to tumor therapy, the magnetic iron oxide-based nanoparticles also offer diagnostic (e.g., MRI) potential [5,6,7]. On the other hand, the gold nanoparticles are known to show a surface plasmon resonance usable in photothermal therapy or diagnostics [8,9,10,11]. Hybrid systems, consisting of gold and iron oxide nanoparticles, offer a combination of these properties within one nanosystem [12], which has been of a great interest in the field of drug delivery agents [13]. In particular, the coating of the magnetic iron oxide nanoparticles with the biocompatible precious metal, gold, prevents not only the chemical and enzymatic degradation of the iron oxide core, but also seems to be suitable for further binding of various types of compounds (especially sulfur-containing ones) and functionalization [14,15].

There are plenty of works, as shown below, reporting gold/iron oxide-based nanoparticles available for further derivatization and/or functionalization of gold. Surprisingly, only one of them describes functionalization with platinum-based species, namely cisplatin [13]. In this case, the activated cisplatin was bound to the thiolated polyethylene glycol (PEG) linker (the thiol part of the linker was lipoic acid (HLA), also used in this work). *In vitro* cytotoxicity of these nanoparticles was found to be more than 100-times higher against both the cisplatin-sensitive and resistant human ovarian carcinoma cancer cell lines (A2780, A2780/cp70) as compared to cisplatin. It can be highlighted that the herein reported mag/Au–LA–CDDP* nanoparticles represent a synthetically more easily and quickly obtainable system (there is no need for the preparation of the thiolated PEG linker), which is (very important from the pharmacological point of view) able to bind even more platinum(II) species (as discussed below).

Regarding other maghemite/gold nanoparticle systems, those layered by differently-thiolated PEG were reported as a promising MRI contrast diagnostic agent for malignant tumors [16]. Similar systems, but with anti-podoplanin antibody bound to PEG on the surface of the studied nanoparticles, were shown as suitable MR imaging agents in evaluating lymphangiogenesis in breast cancer cells *in vivo* [17]. Fan *et al.* prepared gold-coated maghemite core-shell nanoparticles that highly specifically targeted SK-BR-3 human breast cancer cells through the S6 aptamer bound on the surface, which exhibit SK-BR-3 cells targeting themselves [18]. Modification of the surface by fibrinogen resulted in interesting cell targeting properties, representing another possible application of gold-coated iron oxide core-shell nanoparticles [19]. Similar nanoparticles functionalized with different agents (PEG, glucose or fluorochrome) did not show any *in vitro* cytotoxicity against the cervix carcinoma HeLa cancer cell line [20]. Interesting possibilities within this field of study are hybrid nanosystems with an iron oxide core and a gold shell separated by an intermediate layer (e.g., polyethyleneimine) [21] or dumbbell-like nanocomposites consisting of a gold nanoparticle bound together with an iron oxide one, where the gold part can be functionalized by the sulfur-containing molecules or linkers [22].

In this study, we deal with the preparation and characterization of potential theranostic maghemite/gold nanoparticles layered with a simple sulfur-containing carboxylic acid (lipoic acid) and directly functionalized by the platinum metallotherapeutic-based species, such as the herein used activated cisplatin. The reported data represent an optimized and easily realized pathway leading to gaining of the discussed biocompatible nanosystem, which may serve as a necessary base for further biological studies.

## 2. Results and Discussion

### 2.1. Preparation and Functionalization

**Figure 1:**
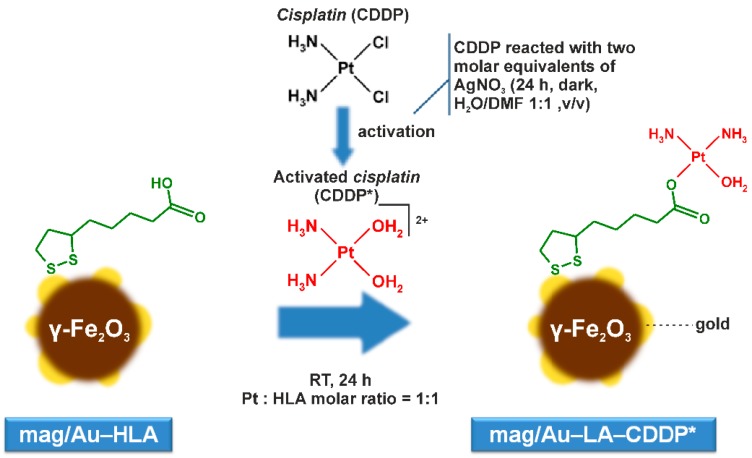
The reaction pathway leading to the preparation of the mag/Au–LA–CDDP* nanoparticle system.

The synthesis of the magnetite nanoparticles, maghemite nanoparticles, iron oxide/gold nanoparticles system, as well as the iron-oxide/gold nanoparticle hybrid system layered by organic sulfur-containing carboxylic acid is well-established in the literature [13,23,24,25]. In the case of this work, the freshly prepared magnetite nanoparticles were gently oxidized by diluted nitric acid to produce the maghemite nanoparticles (mag), which are macroscopically pronounced by the color change from black to brown. The coating of maghemite by gold was carried out by the slow addition of a 1% HAuCl_4_ water solution, again connected with a color change of the nanoparticles (mag/Au) to dark purple (further addition of the HAuCl_4_ solution led to the formation of gold nanoparticles, observable as a purple/pink color above the mag/Au nanoparticles). The prepared mag/Au nanoparticles kept their magnetic properties (being attracted by the external magnet) and are stable in the tetramethylammonium hydroxide (TMAOH) solution for at least two weeks (there are no color changes of the nanoparticles themselves nor the solution above the nanoparticles). As is frequently described in the literature [13,25], gold nanoparticles can easily interact with sulfur-containing molecules. We let a slight excess of lipoic acid (HLA) react with mag/Au, resulting in mag/Au–HLA nanoparticles. Within the final step of the synthetic procedure, including the crucial functionalization, the mag/Au–HLA nanoparticles interacted with the activated platinum-based drug, cisplatin, *i.e.*, with *cis*-[Pt(NH_3_)_2_(H_2_O)_2_]^2+^ species (CDDP*) prepared by the reaction of cisplatin (*cis*-[Pt(NH_3_)_2_Cl_2_], CDDP) with two molar equivalents of AgNO_3_ in the dark [26], to give the final product of the mag/Au–LA–CDDP* system (Figure 1). Regarding the yield of the synthesis, we got 155 mg of mag/Au–LA–CDDP*, but this cannot be expressed as a percent, because every synthetic intermediate was partly used for synthesis and partly stored or used for characterization.

Regarding the lipoic acid used, it has to be stated that it was previously used, for example, for the modification of the gold seeds of the iron oxide magnetic core, where lipoic acid was subsequently modified by the interaction with iminodiacetic acid, which finally bound copper(II) ions [27], or lipoic acid was used as the thiol part of the thiolated polyethylene glycol (PEG) linker layering the gold-coated maghemite nanoparticles and binding the activated cisplatin as functionalizing platinum-based species [13]. In comparison to the latter work, here, we applied a different approach resulting in the magnetic gold-coated maghemite nanoparticles layered by lipoic acid itself, with its carboxylic group ready for the covalent binding of the biologically-active platinum-based species, such as activated cisplatin. Interestingly, the use of the lipoic acid itself (this work), instead of the LA–PEG thiolated polyethylene glycol linker [13], not only simplified and accelerated the synthesis, but also provided the nanosystems with a higher Pt content (as discussed below).

### 2.2. Characterization

#### 2.2.1. HRTEM, TEM and SEM Microscopy

**Figure 2:**
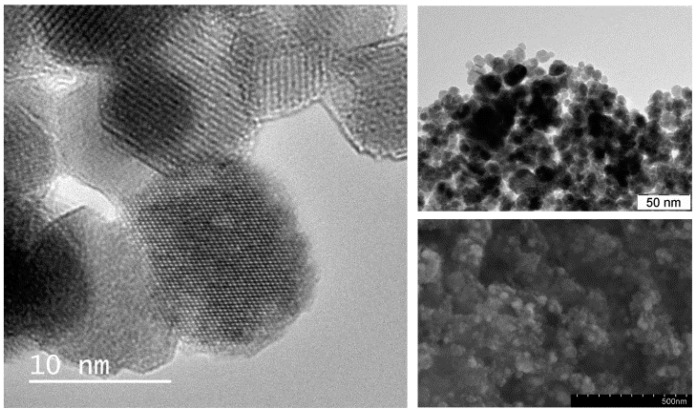
HRTEM (**left**; 10 nm size bar), TEM (**top right**; 50 nm size bar) and SEM (**bottom right**; 500 nm size bar) images, as obtained for the studied functionalized mag/Au–LA–CDDP* nanoparticles.

The prepared mag/Au–LA–CDDP* nanosystems are of a uniform spherical shape (average size of 12.2 ± 1.9 nm), as proven by the HRTEM, TEM and SEM techniques applied to characterize their morphology and dispersibility (Figure 2). Any impurity (e.g., crystalline lipoic acid or CDDP*) was not microscopically detected within the studied product. The intermediates, mag, mag/Au and mag/Au–HLA, showed similar morphological properties compared with the discussed mag/Au–LA–CDDP*. The dispersibility of all of the intermediates and the final functionalized nanoparticles was comparable, as well, with no agglomeration observed after the individual synthetic steps.

**Figure 3:**
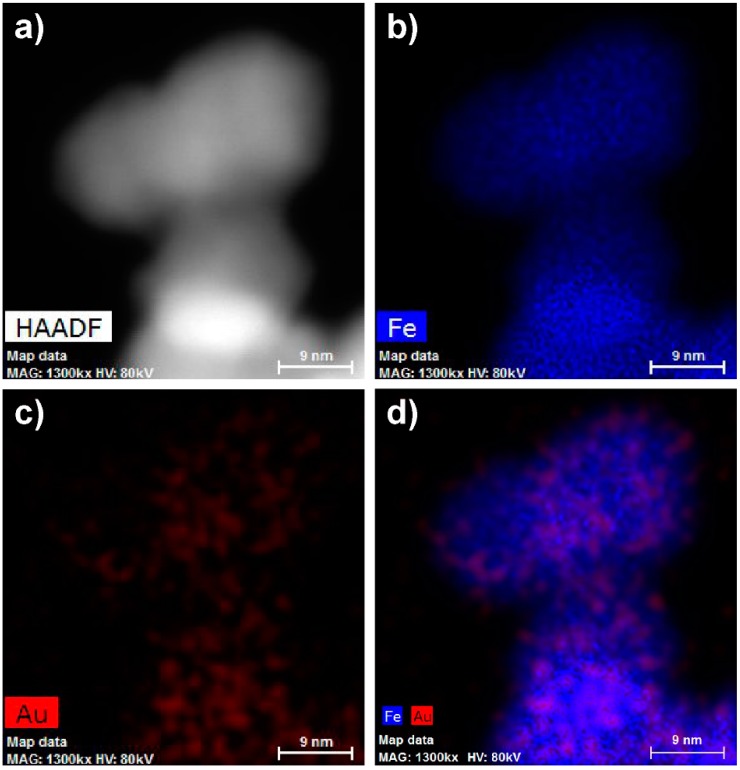
High-angle annular dark-field detector (HAADF)-STEM images of mag/Au nanoparticles (**a**–**d**; 9 nm size bars) showing iron (**b**), gold (**c**) and both iron and gold present in one nanoparticle (**d**).

Although we applied the synthetic procedure reported for the preparation of the gold-coated maghemite core-shell nanoparticles, we did not detect gold as a compact layer coating the surface of the maghemite, but more or less randomly distributed on the magnetic iron oxide support (Figure 3).

#### 2.2.2. XPS Spectroscopy

**Figure 4:**
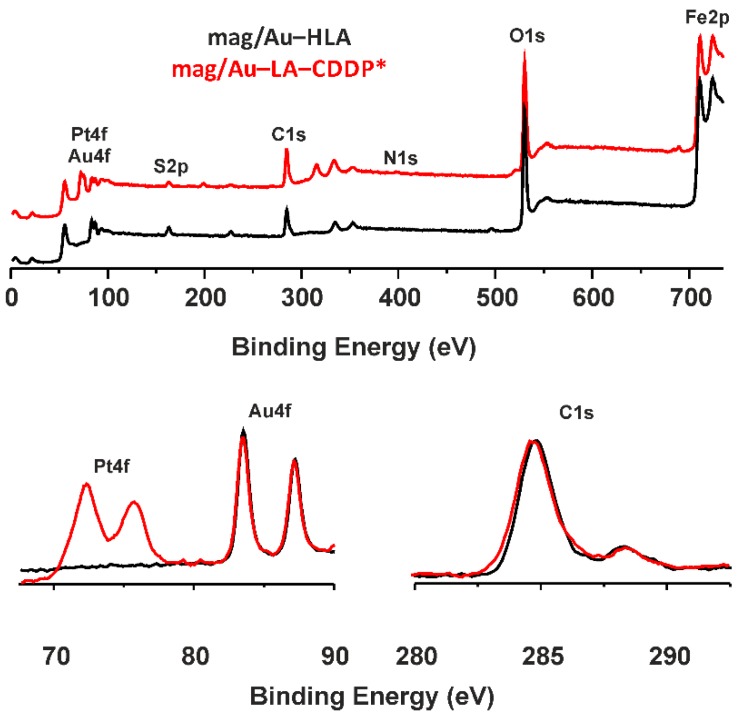
The results of the XPS spectroscopy of the mag/Au–LA–CDDP* nanosystems (red lines) and their comparison with mag/Au–HLA (black lines), given for the 0–750 eV region (**top**) with the details of the Pt4f/Au4f region at 67.5–90 eV (**bottom left**) and C1s region in the 280–295 eV range (**bottom right**).

The XPS spectra were recorded for the final mag/Au–LA–CDDP* nanosystem and its synthetic precursor, mag/Au–HLA (Figure 4). As can be anticipated, the XPS spectra of both systems were similar (the data given below belong to mag/Au–LA–CDDP*). The photoelectron peaks assignable to the maghemite part of the composite were at 711.0 and 724.2 eV (Fe2p_3/2_ and Fe2p_1/2_) and 530.2 eV (O1s) [24,28]. The spectra also contained several gold photoelectron peaks, namely Au4p_3/2_ (353.8 eV), Au4p_5/2_ (333.4 eV), Au4f_5/2_ (87.0 eV) and Au4f_7/2_ (83.8 eV) [29,30,31]. Similar data were also reported for the gold nanoparticles [32] or gold seeds on the surface of the magnetite nanoparticles [27], which were covalently layered with the herein used lipoic acid. As for the lipoic acid itself, the peaks of C1s at 284.6 eV (aliphatic chain carbon atoms) and 288.4 eV (carboxylic group carbon atoms), as well as the S2p peak (163.0 eV) have to be considered as evidence of the lipoic acid incorporation into the studied nanoparticles [27,33].

The differences between the XPS spectra of mag/Au–HLA and mag/Au–LA–CDDP*, clearly showing for the CDDP* the presence within mag/Au–LA–CDDP*, can be found at the 399.8 eV region characteristic for N1s peaks and mainly in the regions connected with platinum, whose photoelectron peaks were detected at binding energies of 315.8 (Pt4d_5/2_), 75.7 (Pt4f_5/2_) and 72.3 (Pt4f_7/2_) eV (Figure 4) [24,34,35]. The position of the Pt4f photoelectron peaks within the XPS spectrum of mag/Au–LA–CDDP* indicated that platinum keeps the +II oxidation state, because it falls between Pt(0) (74.5 and 71.0 eV) and Pt(IV) (78.0 and 74.5 eV) [34]. Further, the position of the Pt4f and N1s peaks correlated well with those reported for cisplatin, *cis*-[Pt(NH_3_)_2_(H_2_O)Cl]^+^ or activated cisplatin bound to mercaptosuccinic acid on the surface of the functionalized hydroxyapatite nanoparticles [34,35].

#### 2.2.3. EDS Spectroscopy

**Figure 5:**
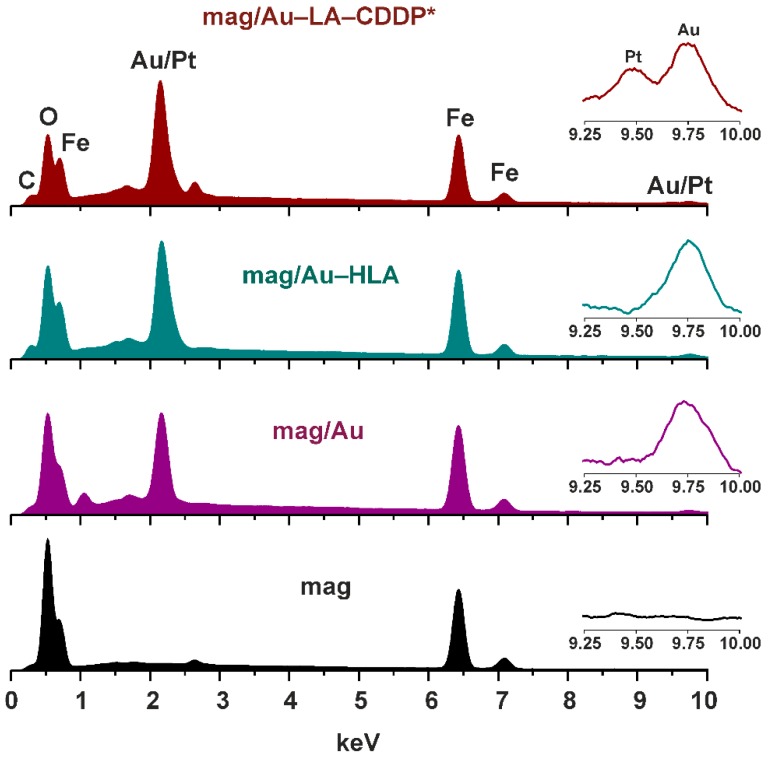
EDS spectra of the final functionalized mag/Au–LA–CDDP* nanoparticles and their synthetic intermediates, mag/Au–HLA, mag/Au and mag nanoparticles, with the assigned peaks of carbon, oxygen, iron and gold/platinum. Insets: detail of the gold/platinum region (depicted in the 9.25–10.00 keV range).

The EDS spectra were recorded for the products of all of the synthetic steps (mag, mag/Au, mag/Au–HLA, mag/Au–LA–CDDP*) (Figure 5).

The discussed XPS results correlated very well with those obtained by the EDS spectroscopy and nicely reflected the synthetic procedure. Particularly, coating of mag nanoparticles, whose EDS contained only the Fe (0.70, 6.43 and 7.08 keV) and O (0.53 keV) peaks, with gold resulted in the Au peaks detected in the EDS spectrum of mag/Au nanocomposites at 1.67, 2.15 and 9.75 keV. Interaction of the mag/Au system with lipoic acid was shown as a new peak at 0.31 keV, a characteristic region for carbon. All of the mentioned peaks were also found in the EDS spectrum of the functionalized mag/Au–LA–CDDP* nanoparticles together with one new peak at 9.49 keV, unambiguously assignable to platinum. The other platinum peaks were not detected in the spectrum of the functionalized nanoparticles, because they are overlapped by the mentioned Au peaks at 1.67 and 2.15 keV.

#### 2.2.4. Quantification by ICP-MS

The platinum and gold contents within the mag/Au–LA–CDDP* nanoparticles were determined by ICP-MS. The results showed that the mentioned functionalized nanoparticles contain platinum and gold in a molar ratio of 1:2.6. Related to the weight of mag/Au–LA–CDDP*, the weight content of platinum is 4.0% (10.6% for gold). Expressed in mol per 1 g of Au, the Pt content within the studied mag/Au–LA–CDDP* nanoparticles equals 1.97 × 10^−3^ mol per 1 g of Au, which is a *ca*. 2.5-times higher Pt-to-Au ratio compared with the similar previously reported maghemite/gold nanoparticles layered by PEG thiolated by lipoic acid and functionalized with the activated cisplatin (7.9 × 10^−4^ mol per 1 g of Au) [13].

#### 2.2.5. Simultaneous TG/DTA Thermal Analyses

**Figure 6:**
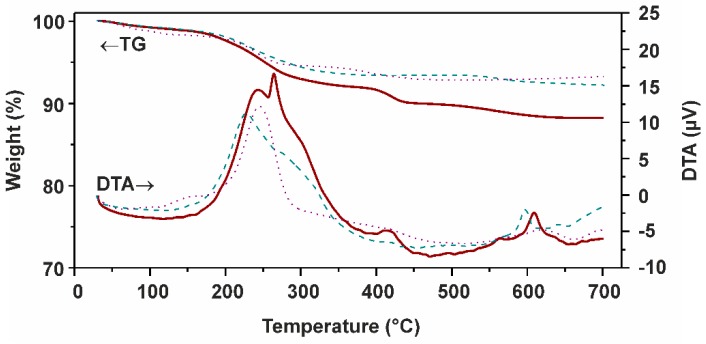
Thermogravimetry (TG) and differential thermal analysis (DTA) results obtained for mag/Au–LA–CDDP* (red curves), mag/Au–HLA (dashed green curves) and mag/Au (dotted purple curves).

A comparison of the results of the simultaneous TG/DTA thermal analyses showed the considerable differences between mag/Au–LA–CDDP* nanoparticles and their synthetic intermediates mag/Au–HLA and mag/Au (Figure 6). As for mag/Au, we detected the usual weight loss (30–145 °C) connected with the loss of physically adsorbed water [28,36], which fluently continued up to 508 °C by the next weight loss, which is, as supported by a massive exothermic effect (maximum at 243 °C), most probably connected with an oxidation of the remaining citrate on the surface of the mentioned nanoparticles (the presence of the citrate within mag/Au can be supported by the EDS results, which showed the Na peak at 1.05 keV; Figure 5) [37]. In the case of mag/Au–HLA, there are two steps following the above-mentioned initial water loss, detected at 101–413 °C and above 496 °C, connected with the decomposition of the organic layer (lipoic acid) of these nanoparticles. However, a sharp exothermic peak (maximum at 597 °C) is not connected with this process, but it has to be assigned to the γ-Fe_2_O_3_ to α-Fe_2_O_3_ conversion [36].

The final functionalized mag/Au–LA–CDDP* nanoparticles showed a similar TG curve, but with a marked weight loss at 367–465 °C accompanied by an exothermic effect with a maximum at 416 °C, which most probably relates to the platinum-containing species decomposition. Again, the exothermic effect of the γ-Fe_2_O_3_ to α-Fe_2_O_3_ conversion was found on the DTA curve at 609 °C. Another important finding comes from the sharp exothermic effect detected on the DTA curve of mag/Au–LA–CDDP* at 263 °C, which can be assigned to the *cis*-to-*trans* rearrangement of the platinum-containing species, which showed that a Pt(NH_3_)_2_ motif binds to one LA through one Pt–O band; in other words, only one H_2_O ligand of the interacting *cis*-[Pt(NH_3_)_2_(H_2_O)_2_]^2+^ species was replaced by the O-donor, lipoic acid.

#### 2.2.6. IR Spectroscopy

**Figure 7:**
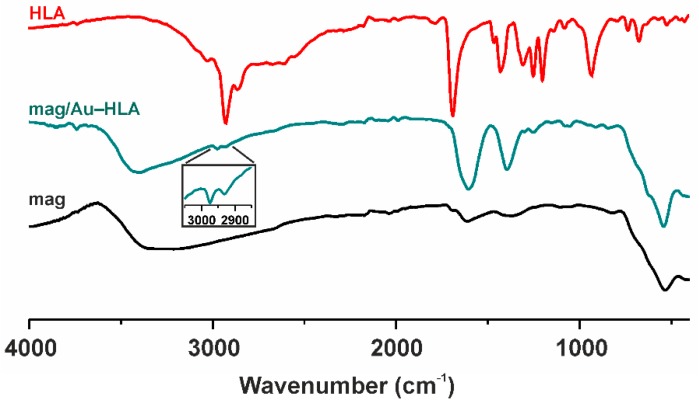
The IR spectra of the mag/Au–HLA nanoparticles (green; the detail of the aliphatic C–H vibration region is given as an inset) and its comparison with the mag nanoparticles (mag; black) and free lipoic acid (HLA; red).

The IR spectrum of mag/Au–HLA is depicted, together with the spectra of mag nanoparticles and free HLA, in Figure 7. Both the spectra of mag/Au–HLA and mag nanoparticles contained peak at *ca.* 540 cm^−1^, clearly assignable to the ν (Fe–O) vibration of maghemite [24,38]. In the spectrum of mag/Au–HLA, we also detected a series of peaks revealed at 1604 and 1387 cm^−1^, which may be associated with the C=O and C–O stretching vibrations, respectively, and the peaks at 2931 and 2974 cm^−1^ corresponding to the stretching vibrations of aliphatic C–H bonds. The peaks observed at *ca.* 920 and 629 cm^−1^ could be connected with the deformation O–H vibrations and stretching C–S vibrations, respectively. These results can support the presence of HLA in the discussed mag/Au–HLA nanoparticles, since their positions correlate well with the positions of the peaks in the spectrum of free lipoic acid [39,40].

2.2.7. ^57^Fe Mössbauer Spectroscopy

**Figure 8:**
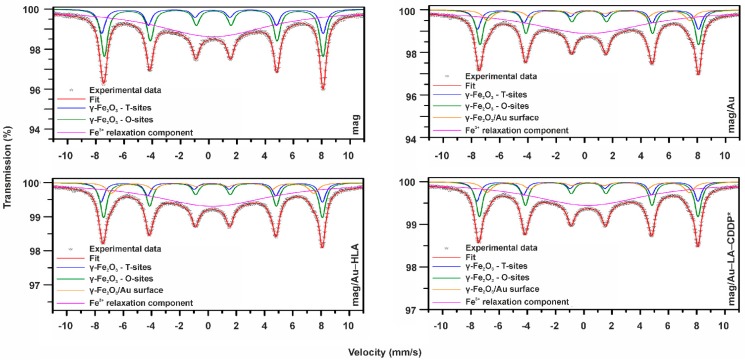
^57^Fe Mössbauer spectrum of the mag, mag/Au, mag/Au–HLA and mag/Au–LA–CDDP* nanoparticles, measured at 300 K and without an external magnetic field.

In order to gain deeper insight into the structural and magnetic properties of the studied samples, ^57^Fe Mössbauer spectroscopy was employed. In Mössbauer spectroscopy, the Fe nucleus acts as a probe monitoring the physicochemical characteristics of the local surroundings through the hyperfine interactions of an electromagnetic nature. The measured ^57^Fe Mössbauer spectra of the studied samples are shown in Figure 8, and the values of the Mössbauer hyperfine parameters, derived from the fitting of the respective Mössbauer spectrum, are listed in Table 1.

**Table 1:** Values of the Mössbauer hyperfine parameters for maghemite (mag), maghemite/gold (mag/Au), maghemite/gold hybrid nanoparticle system layered by lipoic acid (mag/Au–HLA) and maghemite/gold nanoparticles layered by lipoic acid and functionalized by activated cisplatin (mag/Au–LA–CDDP*) derived from the fitting procedure of the respective 300-K Mössbauer spectrum. δ is the isomer shift; Δ*E*_Q_ is the quadrupole splitting; *B*_hf_ is the hyperfine magnetic field; RA is the relative spectral area of individual spectral components.

Sample	Component	δ ± 0.01 (mm/s)	Δ*E*_Q_ ± 0.01 (mm/s)	*B*_hf_ ± 0.3 (T)	RA ± 1 (%)	Assignment
mag	Sextet	0.26	0.01	48.7	13	γ-Fe_2_O_3_–T-sites
	Sextet	0.35	0.00	48.2	25	γ-Fe_2_O_3_–O-sites
	Singlet	0.31	-	-	62	Fe^3+^ relaxation
mag/Au	Sextet	0.27	0.00	48.7	11	γ-Fe_2_O_3_–T-sites
	Sextet	0.37	0.01	48.3	20	γ-Fe_2_O_3_–O-sites
	Sextet	0.31	0.03	44.5	9	γ-Fe_2_O_3_/Au surface
	Singlet	0.31	-	-	60	Fe^3+^ relaxation
mag/Au–HLA	Sextet	0.25	0.01	48.6	11	γ-Fe_2_O_3_–T-sites
	Sextet	0.37	0.01	48.2	20	γ-Fe_2_O_3_–O-sites
	Sextet	0.31	0.04	44.4	9	γ-Fe2O3/Au surface
	Singlet	0.31	-	-	60	Fe^3+^ relaxation
mag/Au–LA–CDDP*	Sextet	0.26	0.00	48.8	11	γ-Fe_2_O_3_–T-sites
	Sextet	0.36	0.00	48.3	20	γ-Fe_2_O_3_–O-sites
	Sextet	0.32	0.04	44.3	9	γ-Fe_2_O_3_/Au surface
	Singlet	0.31	-	-	60	Fe^3+^ relaxation

The slightly asymmetric profile of the room-temperature ^57^Fe Mössbauer spectrum of the maghemite (mag) nanoparticles implies the presence of more than one sextet component with similar values of the Mössbauer hyperfine parameters. Thus, to correctly fit the spectrum, the mag nanoparticles were placed in an external magnetic field applied in a parallel direction with respect to the propagation of γ-rays. The in-field room-temperature Mössbauer spectrum of the mag nanoparticles clearly showed two resolved sextets (not shown) with values of the Mössbauer hyperfine parameters typical of γ-Fe_2_O_3_; one sextet corresponding to the tetrahedral Fe^3+^ cation sites (*i.e.*, T-sites) of the γ-Fe_2_O_3_ spinel structure and the other sextet reflecting octahedral Fe^3+^ cation sites (*i.e.*, O-sites) of the γ-Fe_2_O_3_ spinel structure [41]. It is known that in an external magnetic field, the T- and O-related sextets are well separable, due to the addition and subtraction of the external magnetic field for the γ-Fe_2_O_3_ T-sites and O-sites, respectively [41]. In addition, the spectral area of the O-sextet to T-sextet (~1.90) is far from the ideal ratio (~1.66), indicating that γ-Fe_2_O_3_ is not purely stoichiometric. For γ-Fe_2_O_3_ with sizes of less than 20 nm, the non-stoichiometry results from either distribution of vacancies on the T-sites or the presence of some non-oxidized Fe^2+^ ions [41].

However, in the case of mag, both factors may play an equal role, as their signatures cannot be clearly identified in the zero-field and in-field room-temperature Mössbauer spectra. Nevertheless, the knowledge of the values of the Mössbauer hyperfine parameters of the two sextets and their spectral areas, derived from the in-field Mössbauer spectrum, assisted in helping to fit the zero-field Mössbauer spectrum of the mag nanoparticles. Besides the two sextets ascribed to cation T-sites and O-sites in the γ-Fe_2_O_3_ spinel structure, an extra component, a broad singlet, emerges (see Figure 8). It has features of the Fe^3+^ relaxation component and indicates an onset of the passage of nanoparticle superspin to the superparamagnetic regime at the timescale of the Mössbauer technique [41]. On the other hand, the clearly evolved sextet components imply that superspins of a fraction of γ-Fe_2_O_3_ nanoparticles (*i.e.*, the largest one) still remain in the magnetically blocked state at 300 K with respect to the characteristic measuring time of the Mössbauer technique. The coexistence of sextets and singlet reflects the particle size distribution in the system, as the passage to the superparamagnetic state is, at a given temperature and measuring time, governed by the nanoparticle size [41]. As the spectral area of a singlet is dominant, most of the magnetic nanoparticles in the assembly likely behave in the superparamagnetic manner.

The room-temperature Mössbauer spectrum of the maghemite/gold nanoparticles (mag/Au) shows four spectral components (see Figure 8), *i.e.*, three sextets and one singlet. The sextets with the higher values of the hyperfine magnetic fields can be again ascribed to the cation T-sites and O-sites in the γ-Fe_2_O_3_ spinel structure, and the singlet corresponds to those γ-Fe_2_O_3_ nanoparticles entering the superparamagnetic regime. In addition, one more sextet is clearly identified; its values of the Mössbauer hyperfine parameters reflect the local surrounding of Fe^3+^ ions, however, affected by the presence of non-magnetic and/or diamagnetic atoms. As pure gold is weakly diamagnetic, it acts as a shield, decreasing the magnetic field in which it is placed. Since a much lower value of the hyperfine magnetic field is observed for the third sextet (see Table 1), it can be explained by partially covering the surface of γ-Fe_2_O_3_ nanoparticles with gold, inducing further weakening of the magnetic interactions among the Fe^3+^ magnetic moments in the surface layers of γ-Fe_2_O_3_ nanoparticles. The interaction of gold with the surface of γ-Fe_2_O_3_ nanoparticles is purely non-covalent, as no change in the isomer shift value of the interaction sextet is observed.

The room-temperature Mössbauer spectrum of the mag/Au–HLA and final functionalized mag/Au–LA–CDDP* nanoparticles resembles that of the mag/Au sample (see Figure 8 and Table 1), implying that both lipoic acid and activated cisplatin (CDDP*) do not influence the surface state of the γ-Fe_2_O_3_ nanoparticles. The spectra show only a smaller signal-to-noise ratio, most probably due to firmer embedding of γ-Fe_2_O_3_ nanoparticles in the organic medium.

#### 2.2.8. Stability Studies

The stability of the mag/Au–LA–CDDP* nanoparticles was investigated in three buffers at different pH values and time points (Table 2).

**Table 2:** Pt content (µg) at different times and pH values for the functionalized mag/Au–LA–CDDP* nanoparticles dispersed in phosphate, carbonate and acetate buffers, incubated at 37 °C for 24, 48 and 72 h and checked by ICP-MS from the supernatant collected over the remnant nanoparticles attracted to the magnet.

Solvent (pH)	Incubation Time		
	24 h	48 h	72 h
Acetate buffer (5.0)	2.5 ± 0.1	1.7 ± 0.3	1.6 ± 0.2
Phosphate buffer (7.0)	5.9 ± 0.2	5.2 ± 0.2	7.5 ± 0.1
Carbonate buffer (9.0)	27.5 ± 0.2	31.5 ± 0.2	33.7 ± 0.3

It has been found that the stability of the prepared nanocomposites is quite pH dependent, because the content of the released platinum-containing species increased with the pH value of the appropriate buffer. In the case of the acidic acetate buffer, the release of the platinum-containing species is negligible, while at pH 7 (phosphate buffer), it reaches the values acceptable for further biological studies (e.g., *in vitro* cytotoxicity) [24]. However, even a several times higher release of the platinum-containing species was found with the carbonate buffer under basic pH values.

## 3. Experimental Section

### 3.1. Materials and Methods

The starting materials, FeCl_3_∙6H_2_O, FeCl_2_∙4H_2_O, lipoic acid (HLA), 25% NH_4_OH, tetramethylammonium hydroxide (TMAOH), sodium citrate tribasic dihydrate, HAuCl_4_, cisplatin, AgNO_3_ and solvents, were supplied by Sigma‑Aldrich Co. (Prague, Czech Republic) and Acros Organics Co. (Pardubice, Czech Republic) and used as received.

Simultaneous thermogravimetric (TG) and differential thermal (DTA) analyses were performed using an Exstar TG/DTA 6200 thermal analyzer (Seiko Instruments Inc., Chiba, Japan); dynamic air atmosphere (100 mL·min^−1^), 25–700 °C (5.0 °C·min^−1^). Scanning electron microscopy (SEM) was performed by a Hitachi 6600 FEG microscope (5 keV accelerating voltage), together with energy-dispersive X-ray spectroscopy (EDS) (Hitachi, Tokyo, Japan). HRTEM microscopic images were recorded by an FEI Titan 60–300 kV (0.06 nm point resolution) with an X-FEG emission gun, a Cs image corrector and an STEM high-angle annular dark-field detector (HAADF) (FEI, Hillsboro, OR, USA). The elemental mapping was carried out by STEM-energy dispersive X-ray spectroscopy (EDS). Transmission electron microscopy (TEM) images were taken on a JEOL 2010 microscope (200 kV, 1.9 Å point-to-point resolutions; JEOL, Peabody, MA, USA). A drop of high-purity water with the ultrasonically dispersed samples was placed onto a holey-carbon film supported by a copper-mesh TEM grid and dried in air at room temperature. The diameter of the nanoparticles was measured by ImageJ software (ImageJ, Bethesda, MD, USA). X-ray photoelectron spectroscopy (XPS) results were obtained using a PHI 5000 VersaProbe II device (Physical Electronics, Chanhassen, MN, USA). Infrared spectra (400–4000 cm^−1^ region) were recorded by the ATR technique on a Nexus 670 FT-IR (Thermo Nicolet, Waltham, MA, USA). ^57^Fe Mössbauer spectra were recorded in a transmission geometry employing a Mössbauer spectrometer operating in a constant velocity regime and equipped with a 50 mCi ^57^Co(Rh) source. For in-field ^57^Fe Mössbauer measurement, the maghemite nanoparticles (mag) were placed inside the chamber of the cryomagnetic system (Oxford Instruments, Abingdon, UK); an external magnetic field of 5 T was applied in parallel direction with respect to the propagation of γ-rays. For fitting the Mössbauer spectra, the MossWinn software program was used [42]; prior to fitting, the signal-to-noise ratio was adjusted by the filtering procedures built in the MossWinn software program and by the statistically-based approach developed by Prochazka *et al.* [43]. The isomer shift values are referred to α-Fe at room temperature.

### 3.2. The Platinum and Gold Contents

The mag/Au–LA–CDDP* nanosystems (2.0 mg) were dissolved in 0.1 mL of aqua regia and then 5000× diluted by distilled water. The Pt and Au contents were determined by inductively-coupled plasma mass spectrometry (ICP-MS) with the obtained values corrected for the adsorption effects (ICP-MS spectrometer 7700×, Agilent, Santa Clara, CA, USA).

### 3.3. Stability Studies

The functionalized mag/Au–LA–CDDP* nanoparticles (2.0 mg) were dispersed in 5 mL of phosphate (pH 7.0), carbonate (pH 9.0) and acetate (pH 5.0) buffers and incubated at 37 °C for 24, 48 and 72 h. The content of platinum, showing the ability of the system to release the drug under different conditions, was checked by ICP-MS from the supernatant collected over the remnant nanoparticles attracted to the magnet.

### 3.4. Synthetic Procedures

#### 3.4.1. Maghemite Nanoparticles (mag)

The water (50 mL) solution of the mixture of FeCl_3_∙6H_2_O (2.16 g; 4.0 mmol) and FeCl_2_∙4H_2_O (0.80 g; 2.0 mmol) was stirred under nitrogen gas until the temperature reached 80 °C. After that, 5 mL of 25% NH_4_OH were added to the solution, and the resulting black mixture was intensively stirred for the next 30 min. Thereafter, the obtained magnetite (Fe_3_O_4_) nanoparticles were attracted by the external magnet, washed with water (3 × 10 mL) and 0.01 M HNO_3_ (3 × 10 mL) and stirred in 25 mL of 0.01 M HNO_3_ at 90 °C for 1 h. The prepared maghemite (γ‑Fe_2_O_3_) nanoparticles were magnetically isolated and washed with an adequate amount of deionized water (3 × 10 mL). Part of the product was washed with acetone (3 × 10 mL) and dried under N_2_ atmosphere to be stored in the solid state (in the fridge); another part was dispersed in 0.1 M TMAOH to be stored as a suspension; and the rest was used for the upcoming synthesis.

#### 3.4.2. Maghemite/Gold Nanoparticle System (mag/Au)

Sodium citrate (200 mL of 0.1 M water solution) was added to the water suspension of maghemite nanoparticles (200 mg), and the suspension was stirred at 90 °C for 10 min. One-milliliter aliquots of HAuCl_4_ (1% water solution; 30 mL) were added to this suspension every 5 min with continuous stirring. The prepared mag/Au nanoparticles were, as in the case of maghemite nanoparticles, isolated by the external magnet, washed with deionized water (3 × 10 mL) and either washed with 3 × 10 mL of acetone and dried under a N_2_ atmosphere to be stored in the solid state, dispersed in 0.1 M TMAOH to be stored as a suspension or used for the synthesis.

#### 3.4.3. Maghemite/Gold Nanoparticles Layered by Lipoic Acid (mag/Au–HLA)

The mag/Au nanoparticles (20 mL of the TMAOH suspension) were attracted by the external magnet, the TMAOH poured out and distilled water (20 mL) poured in. The lipoic acid (HLA; 200 mg) was poured in about a 1.25 molar excess (related to gold) into the suspension, which was intensively stirred overnight. The obtained mag/Au–HLA nanoparticle suspension was split into two portions, *i.e.*, one was isolated as the solid state product and the second one as the TMAOH suspension, as described above for both the maghemite and mag/Au nanoparticles.

#### 3.4.4. Maghemite/Gold Nanoparticles Layered by Lipoic Acid and Functionalized by Activated Cisplatin (mag/Au–LA–CDDP*)

The final step of the synthesis involves the reaction of the mag/Au–HLA nanoparticles (those from the TMAOH suspension with TMAOH replaced by distilled water) with the *cis*-[Pt(NH_3_)_2_(H_2_O)_2_]^2+^ species (CDDP*), which formed from cisplatin (*cis*-[Pt(NH_3_)_2_Cl_2_], CDDP; 132 mg to keep the 1:1 molar ratio with HLA) by its activation (24 h, in the dark, water/DMF 1:1 *v*/*v*) with two molar equivalents of AgNO_3_ (149 mg).

The suspension of mag/Au–HLA with the activated cisplatin (CDDP*) was stirred for 24 h followed by magnetic isolation, washing (3 × 5 mL of DMF, 3 × 5 mL of water and 3 × 5 mL of acetone) and drying under a nitrogen gas flow. The obtained product, mag/Au–LA–CDDP* nanoparticles, was stored in the solid state in the fridge.

## 4. Conclusions

Efficient synthesis of a maghemite/gold hybrid nanoparticle system, as a possible magnetic carrier for the transport of platinum-based metallotherapeutics, is described. Easily obtainable superparamagnetic maghemite/gold-lipoic acid (mag/Au–HLA) nanoparticles, consisting of the maghemite/gold particles layered by a sulfur-containing carboxylic acid (lipoic acid), were found (by HRTEM and TEM) to be of a spherical shape and to have an average size of about 12 nm in diameter. This system is suitable for the binding of the platinum-containing species, particularly activated cisplatin of the composition *cis*-[Pt(NH_3_)_2_(H_2_O)_2_]^2+^. The arrangement of gold on the surface of the maghemite nanoparticles can be indirectly supported by the weakening of the magnetic interactions among the Fe^3+^ magnetic moments, which is probably caused by non-covalent interactions of the maghemite core with the gold situated on its surface. The organic layer, represented by lipoic acid, as well as the incorporation of the functionalizing platinum-containing species did not cause an additional alteration of the magnetic interactions. The platinum-containing species binds monofunctionally (*i.e.*, through one Pt–O bond) through the carboxylic groups of mag/Au–HLA within the final mag/Au–LA–CDDP* nanosystem. The incorporation of the activated cisplatin within the final functionalized mag/Au–LA–CDDP* nanoparticles was proven by XPS and EDS spectroscopy and quantified by ICP-MS as being 4.0% in terms of the platinum content related to the total weight of mag/Au–LA–CDDP*. The stability of the mag/Au–LA–CDDP* nanoparticles in solution was found to be more pH dependent than time dependent, *i.e.*, the highest platinum content released from the nanoparticles was determined under basic conditions (pH 9), but acceptable platinum content was detected also in the phosphate buffer of the physiologically more relevant pH (7.0).
